# Supplementary material for: The local density of optical states of a metasurface
Source: Sci Rep. 2016 Feb 12;6:20655. doi: 10.1038/srep20655 (PMC4751612; doi:10.1038/srep20655)
Supplement: Supplementary Information [file srep20655-s1.pdf]

Supplementary Material: The local density of optical states of a metasurface

Per Lunnemann
DTU Fotonik, Department of Photonics Engineering, Østedsplads 343, DK-2800, Denmark

A. Femius Koenderink
Center for Nanophotonics, FOM Institute AMOLF,
Science Park 104, 1098 XG Amsterdam, The Netherlands
(Dated: October 2, 2015)

SUMS OF MAGNETO-ELECTRIC DYADIC GREENS FUNCTION

The sum presented in equation (5), requires special attention since it converges poorly. The problem has been treated extensively in ref. 1 and utilizes a technique pioneered by P. Ewald. The technique consists in splitting a poorly convergent sum into two convergent terms, $\overleftrightarrow{\mathcal{G}}^{(1)}$ and $\overleftrightarrow{\mathcal{G}}^{(2)}$, which are exponentially convergent. Specifically, considering the sum

$$\Gamma(\boldsymbol{k}_{||},\boldsymbol{r}) = \sum_{m,n} G^0(\boldsymbol{R}_{mn}-\boldsymbol{r})e^{i\boldsymbol{k}_{||}\cdot\boldsymbol{R}_{mn}} \tag{S1}$$

where the scalar Green function is

$$G^0(\boldsymbol{R}_{mn}-\boldsymbol{r}) = \frac{e^{ik|\boldsymbol{R}_{mn}-\boldsymbol{r}|}}{|\boldsymbol{R}_{mn}-\boldsymbol{r}|}. \tag{S2}$$

we may rewrite this as

$$\sum_{m,n}\frac{e^{ik|\boldsymbol{R}_{mn}-\boldsymbol{r}|}}{|\boldsymbol{R}_{mn}-\boldsymbol{r}|}e^{i\boldsymbol{k}_{||}\cdot\boldsymbol{R}_{mn}} = \Gamma^{(1)}+\Gamma^{(2)}. \tag{S3}$$

Here

$$\Gamma^{(1)} = \frac{\pi}{\mathcal{A}}\sum_{\tilde{m}\tilde{n}}\left\{\frac{e^{i(\boldsymbol{k}_{||}+g_{\tilde{m}\tilde{n}})\cdot\boldsymbol{r}_{||}}}{k^z_{\tilde{m}\tilde{n}}}\cdot\left[e^{ik^z_{\tilde{m}\tilde{n}}|z|}\mathrm{erfc}\left(\frac{k^z_{\tilde{m}\tilde{n}}}{2\eta}+|z|\eta\right) + e^{-ik^z_{\tilde{m}\tilde{n}}|z|}\mathrm{erfc}\left(\frac{k^z_{\tilde{m}\tilde{n}}}{2\eta}-|z|\eta\right)\right]\right\} \tag{S4a}$$

and

$$\Gamma^{(2)} = \sum_{mn}\left\{\frac{e^{i\boldsymbol{k}_{||}\cdot\boldsymbol{R}_{mn}}}{2\rho_{mn}}\cdot\left[e^{ik\rho_{mn}}\mathrm{erfc}\left(\rho_{mn}\eta+\frac{ik}{2\eta}\right) + e^{-ik\rho_{mn}}\mathrm{erfc}\left(\rho_{mn}\eta-\frac{ik}{2\eta}\right)\right]\right\}, \tag{S4b}$$

where we used $\boldsymbol{r}=(\boldsymbol{r}_{||},z)$, $k=\omega/c$, $k^z_{\tilde{m}\tilde{n}}=\sqrt{k^2-|\boldsymbol{k}_{||}+\overleftrightarrow{g}_{\tilde{m}\tilde{n}}|^2}$, and $\rho_{mn}=|\boldsymbol{R}_{mn}-\boldsymbol{r}_{||}|$. Convergence of equation (S4b) and equation (S4a) follows from the asymptotic expansion of the error function revealing $z\,\mathrm{erfc}(z)\sim\exp(-z^2)$ for $z\to\infty$.[?] The parameter η can be chosen for optimal convergence, and should be set around $\eta=\sqrt{\pi}/a$, where a is the lattice constant. Naturally, the cut off for the summation over m and n must be chosen at least bigger than the number of propagating grating diffraction orders one expects.For our calculations on metamaterials, with essentially no grating orders, i.e., $ka\leq 2\pi$, we already obtained converged lattice sums for $|m,n|\leq 5$.

The dyadic lattice sums in equation (5) are easily generated by noting that the scalar Green function

$$G(\boldsymbol{r},\boldsymbol{r}') = \frac{\exp\left(ik|\boldsymbol{r}-\boldsymbol{r}'|\right)}{|\boldsymbol{r}-\boldsymbol{r}'|} \tag{S5}$$

sets the dyadic Green function via

$$\overleftrightarrow{G}^0(\boldsymbol{r}-\boldsymbol{r}') = \begin{pmatrix}\mathbb{I}k^2+\nabla\otimes\nabla & -ik\nabla\times\\ ik\nabla\times & \mathbb{I}k^2+\nabla\otimes\nabla\end{pmatrix}G(\boldsymbol{r},\boldsymbol{r}') \tag{S6}$$

where $\mathbb{I}$ indicates the 3×3 identity matrix and $\otimes$ denotes the outer product. The derivatives can be simply pulled into each exponentially convergent sum to be applied to each term separately, and are most easily implemented in

practice by noting that the sum $\Gamma^{(2)}$ only depends on radius in spherical coordinates ρ_{mn}, while the sum in $\Gamma^{(1)}$ only depends on radius and height in cylindrical coordinates. For these coordinate systems the differential operator in equation (S6) take particularly simple forms. For spherical coordinates this form reads

$$(\mathbb{I}k^2 + \nabla\nabla)F(r) = \mathbb{I}\left[k^2 F(r) + \frac{1}{r}\frac{d}{dr}F(r)\right] + \begin{pmatrix} x^2 & xy & xz \\ xy & y^2 & yz \\ xz & yz & z^2 \end{pmatrix} \frac{1}{r}\frac{d}{dr}\left[\frac{1}{r}\frac{d}{dr}F(r)\right] \tag{S7a}$$

and

$$-ik\nabla \times F(r) = ik \begin{pmatrix} 0 & z & -y \\ -z & 0 & x \\ y & -x & 0 \end{pmatrix} \frac{1}{r}\frac{d}{dr}F(r), \tag{S7b}$$

which can be directly applied to the summands in equation (S4b). For cylindrical coordinates the differential form reads

$$(\mathbb{I}k^2 + \nabla\otimes\nabla)e^{i\boldsymbol{k}\cdot\rho}g(z) = \begin{pmatrix} k^2 - k_x^2 & -k_x k_y & 0 \\ -k_x k_y & k^2 - k_y^2 & 0 \\ 0 & 0 & k^2 \end{pmatrix} e^{i\boldsymbol{k}\cdot\boldsymbol{r}_{||}} g(z) + \begin{pmatrix} 0 & 0 & ik_x \\ 0 & 0 & ik_y \\ ik_x & ik_y & 0 \end{pmatrix} e^{i\boldsymbol{k}\cdot\boldsymbol{r}_{||}} \frac{\mathrm{d}g(z)}{\mathrm{d}z} + \begin{pmatrix} 0 & 0 & 0 \\ 0 & 0 & 0 \\ 0 & 0 & 1 \end{pmatrix} e^{i\boldsymbol{k}\cdot\boldsymbol{r}_{||}} \frac{\mathrm{d}^2 g(z)}{\mathrm{d}z^2} \tag{S8a}$$

and

$$-ik\nabla \times e^{i\boldsymbol{k}\cdot\boldsymbol{r}_{||}}g(z) = \begin{pmatrix} 0 & 0 & -kk_y \\ 0 & 0 & kk_x \\ kk_y & -kk_x & 0 \end{pmatrix} e^{i\boldsymbol{k}\cdot\boldsymbol{r}_{||}} g(z) + \begin{pmatrix} 0 & ik & 0 \\ -ik & 0 & 0 \\ 0 & 0 & 0 \end{pmatrix} e^{i\boldsymbol{k}\boldsymbol{r}_{||}} \frac{dg(z)}{dz} \tag{S8b}$$

which can be directly applied to evaluate the dyadic equivalent of equation (S4a).

REFERENCES

[1] Linton, C. M. Lattice Sums for the Helmholtz Equation. *SIAM Rev.* 52, 630-674 (2010).
